# National electronic primary health care database in monitoring performance of primary care in Kyrgyzstan

**DOI:** 10.1017/S1463423622000019

**Published:** 2022-02-03

**Authors:** Tiina Laatikainen, Laura Inglin, Ilyas Chonmurunov, Bakhtiar Stambekov, Aliina Altymycheva, Jill L. Farrington

**Affiliations:** 1 Institute of Public Health and Clinical Nutrition, University of Eastern Finland, Kuopio, Finland; 2 Finnish Institute for Health and Welfare, Helsinki, Finland; 3 Joint Municipal Authority for North Karelia Health and Social Services (Siun Sote), Joensuu, Finland; 4 Republican Center for Health, Bishkek, Kyrgyzstan; 5 WHO Country Office of Kyrgyzstan, Bishkek, Kygyzstan; 6 World Health Organization Regional Office for Europe, UN City, Copenhagen, Denmark

**Keywords:** electronic health record, low- and middle-income country, monitoring, noncommunicable disease, primary health care, quality indicator

## Abstract

**Aim:**

The aim of this study was to assess the feasibility of the national electronic primary health care (PHC) database in Kyrgyzstan in producing information on the disease burden of the patient population and on the processes and quality of care of noncommunicable diseases (NCDs) in PHC.

**Background:**

Strengthening of the PHC is essential for low- and middle-income countries (LMICs) to tackle the increasing burden of NCDs. Capacity building and quality improvement require timely data on processes and quality of care.

**Methods:**

A data extraction was carried out covering four PHC clinics in Bishkek in 2019 to pilot the use of the national data for quality assessment purposes. The data included patient-level information on all appointments in the clinics during the year 2018 and consisted of data of altogether 48 564 patients. Evaluation indicators of the WHO Package of Essential NCD Interventions framework were used to assess the process and outcome indicators of patients with hypertension or diabetes.

**Findings:**

The extracted data enabled the identification of different patient populations and analyses of various process and outcome indicators. The legibility of data was good and the structured database enabled easy data extraction and variable formation on patient level. As an example of process and outcome indicators of those with hypertension, the blood pressure was measured at least on two occasions of 90% of women and 89% of men, and blood pressure control was achieved among 61% of women and 53% of men with hypertension. This study showed that a rather basic system gathering nationally patient-level data to an electronic database can serve as an excellent information source for national authorities. Investments should be made to develop electronic health records and national databases also in LMICs.

## Introduction

Health service systems in developing countries are struggling with the weakest and the lowest resourced health systems with underdeveloped primary health care (PHC), while at the same time having the highest burden of noncommunicable diseases (NCDs) within the WHO European Region (Jakab *et al.*, 2018). In Kyrgyzstan, a lower-middle income, former-Soviet-Union country in central Asia, NCDs are the leading cause of mortality and cardiovascular diseases (CVDs) alone cause around half of all deaths (WHO Regional Office for Europe, 2017). An estimated 17% of the population aged 40–64 years is at high CVD risk, and the Ministry of Health (MoH) designated 2019 as the year of Hypertension in an effort to increase the focus on control of this major risk factor (Kaliev *et al*., 2018).

Good health is not only important for individuals, but also for governments being crucial in achieving sustainable development and growth as well as constraining the increment of required resources. Almost 4% of Kyrgyzstan’s gross domestic product is lost due to NCDs. Life expectancy of men is only 67 years of age (women 75 years of age) having major influence on workforce (WHO Regional Office for Europe, 2017). Significant improvements in PHC are thus needed to meet the universal health coverage, one of the principal goals among the Sustainable Development Goals (United Nations Development Programme, 2019; World Health Organization, 2019).

A scoping review by Bitton *et al.* (2019) assessing the PHC system performance in low- and middle-income countries (LMICs) and related further needs in research addressed the lacking evidence on feasible and effective surveillance approaches across PHC systems in LMICs. Especially, the need for further work to develop better NCD surveillance was highlighted. A systematic review by Kruse *et al.* (2018) identified that the use of electronic health records (EHRs) facilitated management of population health by improving productivity and efficiency, improved quality, data management, possibilities for surveillance, and support for preventive care. Most of the research related to the use of EHRs has concentrated on effects on processes and outcomes of care in clinical settings. Much less is known about the feasibility of EHRs in providing large-scale and timely data on health situation of population available for health departments (Klompas *et al.*, 2012).

Very little research is available on how to implement and improve the local use of information communication technology and eHealth to improve the outcomes in PHC in LMICs nor what ideal, low-cost, and simple eHealth documentation and data recording systems exist or could be implemented in low-resource settings (Bitton *et al*., 2019). In Kyrgyzstan, it has been found that quality is driven by a top-down approach rather than inherent in the work of clinical teams, and routinely collected data on the management of patients and facilities are not generally shared nor routinely available for performance feedback to managers or clinicians (World Health Organization, 2018).

The aim of this study was to assess the feasibility of the national electronic PHC database administered by the eHealth Center in Kyrgyzstan to obtain valuable information for the national authorities on the disease burden of the patient population and on the potential of routine data systems to support performance management of NCDs in PHC.

## Material and methods

The national electronic database for PHC data was established in Kyrgyzstan in 2012. The database is administered by the eHealth Center operating under the MoH. The database includes information collected using a clinical information form (CIF) filled in at all visits to PHC clinics. The CIF is a standard form used throughout the country and includes a limited amount of information, but in structural format. Recently, the form was nationally updated to include some key indicators related to the evaluation needs of WHO Package of Essential NCD Interventions (WHO PEN) framework as the lack of information was observed when piloting the WHO PEN protocols in Kyrgyzstan in 2015–2017 (World Health Organization, 2013; Kontsevaya and Farringtom, 2017). In the beginning, the CIF forms were filled in on paper by the health professionals and after the appointment entered to the database by assistants in the clinics. Clinic specific data were then transferred to the national eHealth Center once a month. More recently, the availability of hardware and software has improved and in some clinics the data can be directly entered to the national database by professionals themselves.

To pilot the use of the national data for quality assessment purposes, a data extraction was done by the eHealth Center covering all data until December 2018 covering four PHC clinics in Bishkek. Because the system has not been introduced to all the clinics at the same time, the dates of the first visits varied starting in some clinics already from December 2014. For analyses, we restricted the data to patients over 18 years of age who had visited the clinics during a 1-year period (25 December 2017–25 December 2018) being comparable between the clinics. Every patient has a unique identifier given in the clinic, so we were able to combine the different visits on the patient level inside the clinics, but not between the clinics.

Quality of care indicators were driven from the indicators used in the evaluation of the implementation of the WHO PEN framework which aims at strengthening efficiency and equity of PHC in low-resource settings (Table 1). Similar indicators have been used in various evaluation studies in eastern European and Central Asian countries, where however the data have been collected manually from paper records (Collins *et al.*, 2019; Laatikainen *et al.*, 2020). Both process indicators describing, for example, the measurement activity of risk factors and follow-up of blood pressure, cholesterol, and glucose levels as well as some outcome indicators assessing the achievement of treatment targets were calculated.

**Table 1.** Definition of indicators

Risk factors	WHO/ISH score>20%	Proportions of patients with a total cardiovascular risk score over 20%
	SBP/DBP	Mean based on the most recent systolic/diastolic blood pressure measurement
	FG	Mean based on the most recent fasting glucose measurement
	TC	Mean based on the most recent total cholesterol measurement
**Process indicators**	BP measured regularly	Proportion of patients with two documented blood pressure measurements
	WHO/ISH score documented	Proportions of patients with a documented total cardiovascular risk score
	FG documented	Proportions of patients with fasting glucose tested
	TC documented	Proportions of patients with total cholesterol tested
	HT drug prescribed	Proportions of patients with blood pressure-lowering drug prescribed
Outcome indicators	SBP/DBP < 140/90	Proportion of patients whose last two recorded blood pressure measurements were at normal range (systolic blood pressure < 140 mmHg and diastolic blood pressure < 90 mmHg)
	SBP/DBP < 140/90 (no HT drug)	Proportion of patients with no antihypertensive medication prescribed whose last two recorded blood pressure measurements were at normal range (systolic blood pressure < 140 mmHg and diastolic blood pressure < 90 mmHg)
	SBP/DBP < 140/90 (with HT drug)	Proportion of patients with antihypertensive medication prescribed whose last two recorded blood pressure measurements were at normal range (systolic blood pressure < 140 mmHg and diastolic blood pressure < 90 mmHg)
	SBP/DBP < 130/80	Proportion of diabetic patients whose last two recorded blood pressure measurements were controlled (systolic blood pressure < 130 mmHg and diastolic blood pressure < 80 mmHg)
	FG ≤ 6	Proportion of patients whose last fasting glucose measurement was ≤ 6 mmol/L
	FG < 7	Proportion of diabetic patients whose last fasting glucose measurement was < 7 mmol/L
	TC < 5	Proportion of patients whose last total cholesterol measurement was ≤ 5 mmol/L

WHO/ISH score = World Health Organization/International Society of Hypertension risk prediction score; SBP = systolic blood pressure; DBP = diastolic blood pressure; FG = fasting glucose; TC = total cholesterol; HT = hypertension; BP = blood pressure.

Regarding these key indicators related to NCDs and their management, the following information was available in the database: age, gender, medical diagnosis recorded during the visit (ICD-10), drug prescriptions, total cardiovascular risk assessed using World Health Organization/International Society of Hypertension (WHO/ISH) risk prediction charts, values of blood pressure measurements, fasting glucose, and total cholesterol. However, it seemed that recording of laboratory values (fasting glucose and total cholesterol) had been implemented in the clinics during spring 2018, and thus data from a few first months were missing. Data were checked for implausible values and logical discrepancies. It was found out that the clear structure of the database and the drop-down menus used, for example, in recording medications supported legibility of the data. Obviously, manual data entry is prone to mistakes and such were found especially in recording measurement values. However, the amount of implausible values was actually very small. Another place for error is diagnostic codes where we were able to observe some differences in practices which codes are preferred.

To identify whether patients had diabetes or CVDs, the recordings of diagnoses were considered also from previous years if available. Patients were regarded as having hypertension if ICD-10 codes I10–I15, diabetes if codes E10–E16, ischemic heart disease if codes I20–I25, and cerebrovascular disease if codes I60–I69 were recorded in CIF forms. Other information was taken into account only based on recordings during the 1-year observation period. The most recent values were used to calculate the mean of blood pressure, cholesterol, and fasting glucose. Hypertension medication was identified from the name of the medication prescribed.

Data were analyzed for all patients over 18 years of age as well as separately for those over 40 years of age, for those with hypertension or with diabetes. Differences between men and women were evaluated by logistic regression models for dichotomous outcomes and multivariate linear regression models for continuous outcomes. All results were age-adjusted and a *P*-value < 0.05 was considered statistically significant.

## Results

The characteristics of the patient population that had visited the clinics during the designated period are presented in Table 2. There were altogether 48 564 adult patients who had visited these four clinics during the observed year. Over 70% of them were women, but the age distribution of patients was similar for women and men. Out of all patients, diagnosis for hypertension was recorded for 16% of women and 17% of men, diabetes for 4% and 5%, ischemic heart disease for 9% and 11%, respectively (Table 3). Cerebrovascular disease was found from 8% of both women and men.

**Table 2.** Demographic characteristics of patients

		Total			Hypertensive patients^*^			Diabetic patients^*^		
Characteristic		%	*n*	*N*	%	*n*	*N*	%	*n*	*N*
Gender	Female	71.5	34 742	48 564	69.6	5481	7880	65.2	1220	1871
	Male	28.5	13 822	48 564	30.4	2399	7880	34.8	651	1871
Age, Median (IQR)		39 (28–54)			63 (54–71)			61 (52–69)		
Total, age (years)	18–39	51.1	24 793	48 564	3.4	270	7880	4.8	89	1871
	40–49	16.8	8157	48 564	10.8	851	7880	13.8	259	1871
	50–59	14.2	6894	48 564	25.1	1980	7880	27.2	509	1871
	60–69	10.4	5048	48 564	30.8	2429	7880	31.4	588	1871
	70+	7.6	3672	48 564	29.8	2350	7880	22.8	426	1871
Women, age (years)	18–39	52.3	18 161	34 742	2.9	158	5481	4.4	54	1220
	40–49	16.6	5776	34 742	10.0	550	5481	10.7	131	1220
	50–59	13.3	4627	34 742	23.5	1288	5481	24.0	293	1220
	60–69	10.1	3494	34 742	31.3	1714	5481	32.8	400	1220
	70+	7.7	2684	34 742	32.3	1771	5481	28.0	342	1220
Men, age (years)	18–39	48.0	6632	13 822	4.7	112	2399	5.4	35	651
	40–49	17.2	2381	13 822	12.5	301	2399	19.7	128	651
	50–59	16.4	2267	13 822	28.8	692	2399	33.2	216	651
	60–69	11.2	1554	13 822	29.8	715	2399	28.9	188	651
	70+	7.1	988	13 822	24.1	579	2399	12.9	84	651

* Medical diagnoses from all available years were taken into account.

**Table 3.** Documented diseases and risk factors for women and men by patient group

		Women			Men			Age adjusted *P* for difference
		%/mean	*n*/SD	*N*	%/mean	*n*/SD	*N*	
All patients	Hypertension, (%)	15.8	5481	34 742	17.4	2399	13 822	0.005
	Diabetes, (%)	3.5	1220	34 742	4.7	651	13 822	<0.001
	Ischemic heart diseases, (%)	8.8	3064	34 742	10.6	1460	13 822	<0.001
	Cerebrovascular diseases, (%)	7.9	2737	34 742	8.4	1164	13 822	0.142
	WHO/ISH score >20%, (%)	3.9	471	12 197	5.5	277	5073	<0.001
	SBP mean, (mmHg)	113.1	14.3	28 628	119.0	12.5	11 130	<0.001
	DBP mean, (mmHg)	72.5	9.1	28 628	76.6	7.9	11 130	<0.001
	FG mean, (mmol/L)	4.9	1.45	3087	5.2	1.97	1345	<0.001
	TC mean, (mmol/L)	4.4	0.81	2819	4.4	0.87	1223	0.281
40 years of age or more	WHO/ISH score >20%	5.0	433	8599	7.1	260	3674	<0.001
	SBP mean, (mmHg)	120.7	13.9	13 873	123.6	12.7	6138	<0.001
	DBP mean, (mmHg)	76.7	8.4	13 873	78.8	7.6	6138	<0.001
	FG mean, (mmol/L)	5.0	1.51	2617	5.3	2.07	1126	<0.001
	TC mean, (mmol/L)	4.5	0.83	2424	4.5	0.86	1029	0.528
Hypertensive patients	WHO/ISH score >20%	8.5	312	3668	10.6	165	1557	<0.001
	SBP mean, (mmHg)	128.4	14.1	5338	130.1	14.4	2321	<0.001
	DBP mean, (mmHg)	80.1	8.2	5338	81.6	8.4	2321	<0.001
	FG mean, (mmol/L)	5.1	1.56	1518	5.3	1.8	643	0.112
	TC mean, (mmol/L)	4.6	0.88	1433	4.6	0.93	613	0.474
Diabetic patients	WHO/ISH score >20%	11.0	82	744	12.1	51	422	0.149
	SBP mean, (mmHg)	126.4	13.8	1178	125.6	12.7	622	0.253
	DBP mean, (mmHg)	79.6	8.0	1178	79.7	7.1	622	0.057
	FG mean, (mmol/L)	6.7	2.96	358	8.1	3.46	206	0.004
	TC mean, (mmol/L)	4.6	1.03	329	4.6	1.31	175	0.865

WHO/ISH score = World Health Organization/International Society of Hypertension risk prediction score; SBP = systolic blood pressure; DBP = diastolic blood pressure; FG = fasting glucose; TC = total cholesterol; SD = standard deviation.

As an example of process indicators among all the patients, the proportion of those having blood pressure measured at least on two occasions was 70% for women and 64% of men and of about 70% of those aged 40 years or more. Of those with hypertension, the blood pressure was measured at least on two occasions of 90% of women and 89% of men (Table 4). Total cholesterol and fasting glucose were documented for less than 10% of the whole patient population, for about 15% of those aged 40 years or older and for 26% of patients with hypertension. About 30% of patients with diabetes had a recorded value of fasting glucose and a little more than 25% of patients with diabetes had total cholesterol documented in patient records. Total cardiovascular risk was assessed among 67% of women and 65% of men having hypertension and of 61% of women and 65% of men having diabetes. About 60% of patients with hypertension had prescription for an antihypertensive drug.

**Table 4.** Process and outcome indicators for women and men by patient group

			Women			Men			Age adjusted *P* for difference
			%	*n*	*N*	%	*n*	*N*	
All patients	Process indicator^*^	BP measured regularly	70.1	24 366	34 742	64.0	8850	13 822	<0.001
		WHO/ISH score documented	35.1	12 197	34 742	36.7	5073	13 822	0.130
		FG documented	8.9	3087	34 742	9.7	1345	13 822	0.035
		TC documented	8.1	2819	34 742	8.8	1223	13 822	0.061
	Outcome indicator^*^	SBP/DBP < 140/90	89.4	21 772	24 366	84.4	7466	8850	<0.001
		FG ≤ 6	91.7	2831	3087	87.7	1180	1345	<0.001
		TC < 5	73.7	2077	2819	74.8	915	1223	0.860
40 years of age or more	Process indicator	BP measured regularly	72.1	11 959	16 581	70.9	5097	7190	0.074
		WHO/ISH score documented	51.9	8599	16 581	51.1	3674	7190	0.386
		FG documented	15.8	2617	16 581	15.7	1126	7190	0.972
		TC documented	14.6	2424	16 581	14.3	1029	7190	0.755
	Outcome indicator	SBP/DBP < 140/90	80.0	9563	11 959	75.0	3821	5097	<0.001
		FG ≤ 6	90.8	2375	2617	86.3	972	1126	<0.001
		TC < 5	71.2	1725	2424	72.3	744	1029	0.938
Hypertensive patients	Process indicator	BP measured regularly	89.5	4907	5481	88.5	2123	2399	0.171
		WHO/ISH score documented	66.9	3668	5481	64.9	1557	2399	0.054
		FG documented	27.7	1518	5481	26.8	643	2399	0.334
		TC documented	26.1	1433	5481	25.6	613	2399	0.536
		HT drug prescribed	60.4	3309	5481	61.2	1469	2399	0.049
	Outcome indicator	SBP/DBP < 140/90	61.4	3012	4907	52.9	1123	2123	<0.001
		SBP/DBP < 140/90 (no HT drug)	66.9	1184	1771	61.4	458	746	0.004
		SBP/DBP < 140/90 (with HT drug)	58.3	1828	3136	48.3	665	1377	<0.001
		FG ≤ 6	88.5	1343	1518	85.5	550	643	0.120
		TC < 5	63.1	904	1433	65.7	403	613	0.887
Diabetic patients	Process indicator	BP measured regularly	86.7	1058	1220	80.0	521	651	0.001
		WHO/ISH score documented	61.0	744	1220	64.8	422	651	0.246
		FG documented	29.3	358	1220	31.6	206	651	0.608
		TC documented	27.0	329	1220	26.9	175	651	0.846
		HT drug prescribed	42.9	523	1220	31.6	206	651	0.037
	Outcome indicator	SBP/DBP < 130/80	14.3	151	1058	11.9	62	521	0.021
		FG < 7	60.6	217	358	43.2	89	206	0.003
		TC < 5	64.4	212	329	65.7	115	175	0.756

BP = blood pressure; WHO/ISH score = World Health Organization/International Society of Hypertension risk prediction score; FG = fasting glucose; TC = total cholesterol; HT = hypertension; SBP = systolic blood pressure; DBP = diastolic blood pressure.

* See definitions in Table 1.

In general, 89% of women and 84% of men in patient population had blood pressure under 140/90 mmHg (Table 4). Fasting glucose was normal among 92% of women and 88% of men of whom it had been measured. Similarly total cholesterol was normal in about three-quarters of patients from whom it had been measured. Blood pressure control was achieved among 61% of women and 53% of men with hypertension. Based on fasting glucose, 61% of women with diabetes and 43% of men achieved the treatment target < 7 mmol/L. As the proportion of patients with measurement of total cholesterol or fasting glucose was reasonably low, the assessment of treatment targets is not fully relevant, but as shown by the data it could be done if the coverage of measurements would be better.

## Discussion

In general, the information in the database of the eHealth Center enabled the identification of different patient populations and analyses of various process and outcome indicators on management of NCDs. The database allowed patient-level analyses, the legibility of data was good, and the structured database enabled easy data extraction and variable formation. Regarding the quality of the data, the biggest inaccuracies were the discrepancies in coding practices between physicians which were also reported in a study by Klompas *et al.* (2011) and were observed earlier also in Kyrgyzstan in a quality of care review carried out in 2017 (World Health Organization, 2018). This creates difficulties in determining accurate denominators for the calculation of disease prevalences.

The information produced could be easily used nationally in following the health service use and monitoring of some key performance indicators in the service system. Up to our knowledge, this is the first study reporting such a work and analyses done in LMIC. Also, possibilities for similar analyses are so far scarce as national electronic databases including patient-level data do not exist in LMICs.

The analyses showed that in general blood pressure was measured quite actively among the whole patient population and very actively among patients with a chronic condition like diagnosed hypertension or diabetes. However, according to the national protocol which is based on the WHO PEN protocol, biological risk factors should be actively screened among population aged 40 years or more, and among them the measurement rates for blood pressure were not higher compared with the total patient population and cholesterol and blood glucose were measured only among about 15% of them (World Health Organization, 2013). This type of information is useful when evaluating the screening and treatment processes in the service system. It is worth noticing that our data included information for only 1 year and for fasting glucose and total cholesterol even for a shorter period and thus does not give a fully right picture on the situation as according to the recommendations yearly screenings are not mandatory for patients with low risk.

There is not much data published earlier from Kyrgyzstan, but according to WHO Global Health Observatory estimates, the prevalence of raised blood pressure (SBP ≥ 140 OR DBP ≥ 90) was in 2015 in men aged 18 years or more 27.4% and in women 25.7% and the prevalence of diabetes 9.9% of men and 10.8% of women both being clearly higher compared with the results based on this study (The Global Health Observatory (WHO), 2020a; The Global Health Observatory (WHO), 2020b). However, it is obvious that the prevalence of diagnosed patients based on clinical records differ from the population estimates, especially if based on elevated risk factor levels.

There are some problems and challenges to overcome in embedding information and communication technology (ICT) and EHR systems in clinical care in developing countries. These include hardware and software compatibility, lack of quality control, and antiquated infrastructure (Williams and Boren, 2008). Also in Kyrgyzstan, these challenges were observed as the process of electronic data entry timely at the appointment and transfer to the central database worked only partly and in some clinics, the initial phase of data entry was still performed separately from paper records. In addition to need of human resources, it disallows the professionals to taking advantage of the EHR as they cannot use it for example to search information from earlier visits.

One of the strengths of the national electronic PHC database in Kyrgyzstan is nationally standardized content allowing comparable data analyses from different clinics. This would allow automated EHR-based surveillance increasing the quantity, coverage, and timeliness of available data to health authorities compared with traditional surveillance methods (Klompas *et al*., 2012).

One of the key disadvantages of the data collection process in Kyrgyzstan is that unique identifiers for patients are given on the clinic level. Patients moving to another area or visiting different clinics will get a new identification code. This is the main limit of interoperability of the current system and can cause underestimation of actual visits to services, prescription of medications, and procedures or measurements carried out if patients fragment their care between different service providers (Klompas *et al*., 2012). Assurance of standardization and interoperability of systems are crucial to avoid fragmentation of the information environment (Janett and Yeracaris, 2020; Klompas *et al*., 2012). Especially, in developing countries due to lack of resources and various needs of different organizations and departments, the development of comprehensive ICT systems and universal, standardized EHRs fulfilling all the needs is extremely complicated. Thus, it is common that every department implements their own technology making the communication of systems impossible (Williams and Boren, 2008). This challenge has been avoided in Kyrgyzstan as the current system is nationwide and directed by the MoH following the national eHealth strategy (Government of the Kyrgyz Republic, 2015). Bitton *et al*. (2019) addressed in their scoping review the need for research and development of approaches ensuring the interoperability between data sources, scalable and affordable eHealth approaches and innovations strengthening the information systems without significant needs for changes in infrastructure.

In Kyrgyzstan, the EHRs are currently mainly used to collect data for centralized purposes. However, EHRs have shown to have structural and process benefits also at service provider level and they have been shown to improve the legibility of clinical notes (*Holroyd-Leduc et al.*, 2011). In Kyrgyzstan, the current system could be much more utilized also at the clinic level to support the work of professionals. The quality of care review in Kyrgyzstan already recommended that the Mandatory Health Insurance Fund (MHIF) should use the patient-based data for feedback, learning, and improvement, and that for the purpose national databases should be required to provide either aggregated analyses of the data to individual health facilities (World Health Organization, 2018). That would, however, also need some infrastructure development. A meta-analysis carried out by Campanella *et al.* (2015) suggests that EHRs can increase time efficiency and guideline adherence, reduce medication errors, and adverse drug effects. There is also some evidence that introduction of EHRs support the communication between professionals and patients by e-mail and telephone and thus reduce the demand for primary care office visits (Zhou *et al.*, 2007).

If well established, the EHRs can streamline the measurement and analysis of data on clinical performance metrics. However, that needs properly configured systems, accurately completed data fields, and that the software application supports the measurement and reporting process (Janett and Yeracaris, 2020). This pilot study from Kyrgyzstan also showed that the improvement of data quality would need active use of data, identification of mistakes and discrepancies, and further continuous interaction with professionals and other staff entering the data. Also the contents of data collection and correspondingly the structure of the database should be developed to support both the clinical work and the needs of centralized data collection and monitoring.

Implementation of EHRs in developing countries requires human resources, funds, systematic collection of data, and effective monitoring of the existing system. Governments, health managers, and administrators have to have a strong commitment and make also investments (Williams and Boren, 2008). In addition, health care systems should be advised how to prioritize what kind of data to collect and incorporate to EHRs (Rudin *et al.*, 2020). However, this study increased evidence that useful information for national monitoring can be obtained already with reasonably straightforward solutions if the national agreement, commitment, and guidance are on place.

This pilot study on extracting and analyzing national primary care EHR data from Kyrgyzstan showed that even a rather basic system gathering nationally patient-level data to an electronic database can serve as an excellent information source for national authorities. This study was part of a large project commissioned by WHO to support the MoH and MHIF in developing the evaluation of the service system and especially the quality assessment of PHC and the choice of appropriate performance indicators with special focus on hypertension care. Following the aims of the larger project, the results have been thoroughly discussed with the MoH and MHIF. The project included also training and capacity building of eHealth, academic, and MHIF staff enabling similar data extraction and analyses in future and on a larger scale. Local professionals have already been able to replicate this process with some other clinics.
